# Supplementary material for: Serum and Whole Blood Cu and Zn Status in Predicting Mortality in Lung Cancer Patients
Source: Nutrients. 2020 Dec 27;13(1):60. doi: 10.3390/nu13010060 (PMC7824662; doi:10.3390/nu13010060)
Supplement: Supplementary file 1 [file nutrients-13-00060-s001.zip › SUPPLEMENTARY DATA/supplementary tables S1-2.docx]

Table S1 Median serum and whole blood Cu and Zn concentrations and Cu:Zn ratio in lung cancer subgroups according to different factors, presented as median (Q1–Q3).

| Group | Serum Cu | P | Serum Zn | p | Serum Cu:Zn ratio | p | Whole blood Cu | p | Whole blood Zn | p | Whole blood Cu:Zn | p |
| --- | --- | --- | --- | --- | --- | --- | --- | --- | --- | --- | --- | --- |
| DM | | | | | | | | | | | | |
| Yes (n=23) | 0.96  (0.85–1.37) | 0.693 | 0.90  (0.82–0.97) | 0.546 | 1.07  (0.92–1.65) | 0.922 | 1.07  (0.91–1.26) | 0.677 | 6.92  (6.28–7.54) | 0.211 | 0.14  (0.13–0.20) | 0.569 |
| No (n=130) | 1.00  (0.85–1.21) |  | 0.88  (0.77–1.04) |  | 1.17  (0.90–1.48) |  | 1.05  (0.88–1.25) |  | 6.47  (5.62–7.59) |  | 0.16  (0.13–0.20) |  |
| NLR (arbitrary unit) | | | | | | | | | | | | |
| < 2.36 (n=51) | 1.04  (0.89–1.21) | 0.583 | 0.88  (0.75–0.99) | 0.307 | 1.21  (1.01–1.48) | 0.938 | 1.05  (0.84 – 1.20) | 0.754 | 6.58  (5.88–7.43) | 0.207 | 0.16  (0.13–0.18) | 0.425 |
| ≥ 2.36 (n=51) | 1.02  (0.89–1.41) |  | 0.88  (0.80–1.02) |  | 1.20  (0.92–1.54) |  | 1.04  (0.86–1.31) |  | 6.90  (6.25–7.75) |  | 0.19  (0.14–0.22) |  |
| eGFR (mL/min/1.73m^2^) | | | | | | | | | | | | |
| >90 (n=81) | 1.03  (0.85–1.22) | 0.575 | 0.89  (0.80–1.03) | 0.487 | 1.16  (0.88 – 1.57) | 0.418 | 1.08  (0.90–1.28) | 0.825 | 6.38  (5.56–7.66) | 0.530 | 0.16  (0.13–0.21) | 0.845 |
| ≤90 (n=45) | 1.02  (0.87–1.20) |  | 0.87  (0.73–1.06) |  | 1.22  (0.92–1.65) |  | 1.04  (0.86–1.31) |  | 6.70  (5.87–7.48) |  | 0.15  (0.12–0.19) |  |
| Creatinine (mg/dL) | | | | | | | | | | | | |
| <0.7 (n=49) | 1.06  (0.95–1.26) | 0.090 | 0.89  (0.79–1.04) | 0.638 | 1.20  (1.02–1.59) | 0.139 | 1.08  (0.89–1.31) | 0.052 | 6.28  (5.33–7.66) | 0.158 | 0.18  (0.14–0.22) | 0.130 |
| 0.7-1.2 (n=84) | 0.96  (0.80–1.22) |  | 0.89  (0.77–1.05) |  | 1.09  (0.85–1.48) |  | 1.02  (0.83–1.20) |  | 6.62  (5.87–7.43) |  | 0.16  (0.12–0.19) |  |
| >1.2 (n=11) | 1.04  (0.88–1.41) |  | 0.82  (0.79–1.00) |  | 1.24  (1.08–1.48) |  | 1.26  (1.06–1.51) |  | 7.45  (6.40–8.13) |  | 0.16  (0.15–0.23) |  |
| Age (years) | | | | | | | | | | | | |
| <60 y | 0.95  (0.79–1.22) | 0.263 | 0.88  (0.78–1.03) | 0.828 | 1.08  (0.88–1.39) | 0.241 | 1.11  (0.86–1.34) | 0.348 | 6.70  (5.76–7.61) | 0.904 | 0.16  (0.13–0.21) | 0.337 |
| ≥60 y | 1.09  (0.88–1.22) |  | 0.89  (0.78–1.03) |  | 1.20  (0.92–1.54) |  | 1.04  (0.88–1.24) |  | 6.56  (5.73–7.54) |  | 0.16  (0.12–0.20) |  |
| Smoking status | | | | | | | | | | | | |
| Yes (n=44) | 1.07  (0.89–1.32) | 0.514 | 0.84  (0.80–1.03) | 0.083 | 1.23  (1.01–1.63) | 0.362 | 1.11  (0.92–1.29) | 0.483 | 6.62  (5.88–7.81) | 0.915 | 0.16  (0.13–0.21) | 0.607 |
| Previous (n=70) | 1.01  (0.83–1.22) |  | 0.89  (0.75–1.01) |  | 1.15  (0.92–1.51) |  | 0.98  (0.87–1.23) |  | 6.58  (5.71–7.77) |  | 0.15  (0.12–0.19) |  |
| No (n=40) | 1.06  (0.86–1.23) |  | 0.94  (0.82–1.08) |  | 1.09  (0.84–1.48) |  | 1.01  (0.84–1.28) |  | 6.57  (5.94–7.28) |  | 0.16  (0.13–0.20) |  |
| Alcohol consumption |  |  |  |  |  |  |  |  |  |  |  |  |
| Yes (n=76) | 0.98  (0.84–1.22) | 0.141 | 0.88  (0.80–1.01) | 0.842 | 1.13  (0.88–1.51) | 0.217 | 1.03  (0.88 – 1.25) | 0.725 | 6.89  (6.03–7.54) | 0.336 | 0.15  (0.12–0,19) | 0.273 |
| No (n=78) | 1.03  (0.89–1.35) |  | 0.90  (0.75–1.06) |  | 1.21  (0.92–1.56) |  | 1.04  (0.86 – 1.31) |  | 6.57  (5.61–7.68) |  | 0.16  (0.13–0.20) |  |
| BMI (kg/m^2^) |  |  |  |  |  |  |  |  |  |  |  |  |
| <20 (n=12) | 1.12  (0.91–1.20) | 0.463 | 0.88  (0.80–1.06) | 0.471 | 1.13  (0.92–1.44) | 0.866 | 1.16  (1.00–1.60) | 0.075 | 6.28  (5.27–7.19) | 0.513 | 0.19  (0.11–0.27) | 0.101 |
| 20-24,9 (n=49) | 1.02  (0.85–1.40) |  | 0.89  (0.80–1.04) |  | 1.09  (0.89–1.77) |  | 1.11  (0.91–1.35) |  | 6.52  (5.70–7.70) |  | 0.17  (0.14–0.21) |  |
| ≥25 (n=90) | 1.00  (0.86–1.22) |  | 0.88  (0.75–1.01) |  | 1.20  (0.91–1.48) |  | 1.01  (0.87–1.19) |  | 6.89  (5.90–7.60) |  | 0.15  (0.12–0.19) |  |
| WHR (arbitrary unit) |  |  |  |  |  |  |  |  |  |  |  |  |
| <0.85^W^; <0.90^M^ (n=33) | 1.07  (0.85–1.30) | 0.594 | 0.88  (0.77–1.05) | 0.68 | 1.19  (0.89–1.48) | 0.761 | 1.11  (0.92–1.31) | 0.269 | 6.34  (5.31–7.54) | 0.191 | 0.16  (0.14–0.21) | 0.133 |
| ≥0.85^W^; ≥0.90^M^ (n=111) | 1.00  (0.85–1.22) |  | 0.88  (0.78–1.02) |  | 1.15  (0.88–1.56) |  | 1.01  (0.87–1.24) |  | 6.73  (5.87–7.71) |  | 0.15  (0.12–0.19) |  |

DM, diabetes mellitus; NLR, neutrophil:lymphocyte ratio; eGFR, estimated glomerular filtration rate; BMI, body mass index; WHR, waist-hip ratio;

| Parameter | Cut-off value | AUC (95% CI) | Youden’s index | p |
| --- | --- | --- | --- | --- |
| lung cancer patients in clinical stage I-III vs IV disease | | | | |
| Serum Cu concentration [mg/l] | 1.41 | 0.658 (0.537-0.779) | 0.30 | 0.010 |
| Whole blood Zn concentration [mg/l] | 7.12 | 0.705 (0.599 – 0.811) | 0.35 | <0.001 |
| survival vs non-survival lung cancer patients | | | | |
| Serum Cu concentration [mg/l] | 1.19 | 0.668 (0.586 – 0.750) | 0.29 | <0.001 |
| Serum Cu:Zn concentration [mg/l] | 1.34 | 0.604 (0.518 – 0.690) | 0.17 | 0.018 |
| Whole blood Zn concentration [mg/l] | 7.54 | 0.637 (0.551 – 0.723) | 0.27 | 0.002 |

Table S2 Cut-off values, area under the ROC curves (95% CI) and Youden’s index for parameters significantly differentiated clinical stages I-III vs IV and survival vs non-survival lung cancer patients

AUC, area under curve; CI, confidence interval;
