# Supplementary figures and images for: Serum and Whole Blood Cu and Zn Status in Predicting Mortality in Lung Cancer Patients
Source: Nutrients. 2020 Dec 27;13(1):60. doi: 10.3390/nu13010060 (PMC7824662; doi:10.3390/nu13010060)

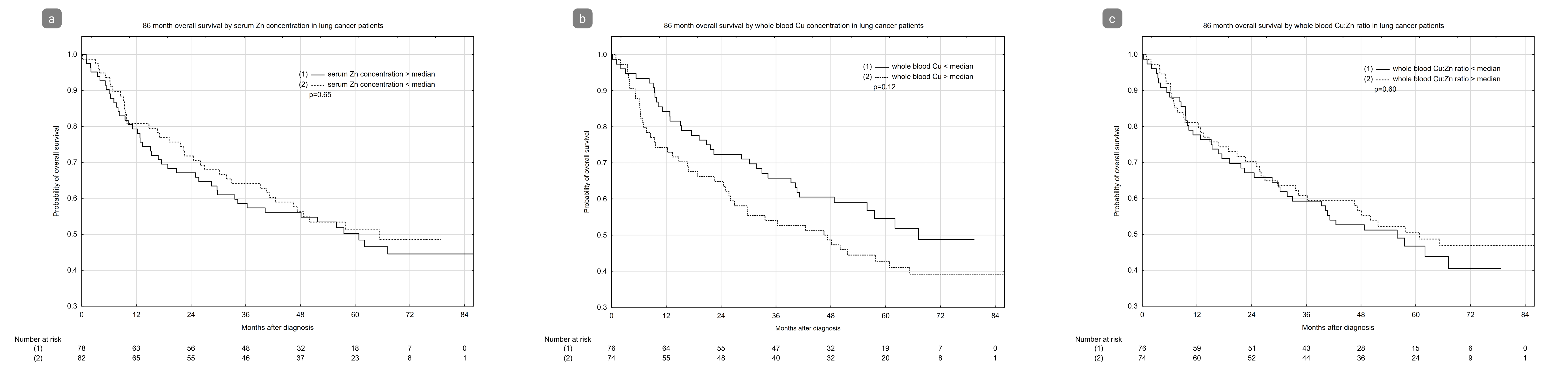

Supplement: Supplementary file 1 [file nutrients-13-00060-s001.zip › SUPPLEMENTARY DATA/Fig_S1.png]
